# Pulsed radiofrequency stimulation suppresses palmar hyperhidrosis in an animal study

**DOI:** 10.1002/brb3.833

**Published:** 2017-09-26

**Authors:** Mu‐Lien Lin, Tzu‐Rung Huang, Ming‐Chien Kao, Hung‐Wei Chiu, Sheng‐Chieh Lin, Fang‐Chia Chang

**Affiliations:** ^1^ Department of Anesthesiology Medical School National Yang‐Ming University Taipei Taiwan; ^2^ Pain Clinic Taipei City Hospital Taipei Taiwan; ^3^ Department of Veterinary Medicine School of Veterinary Medicine National Taiwan University Taipei Taiwan; ^4^ Department of Surgery College of Medicine National Taiwan University Taipei Taiwan; ^5^ Department of Surgery National Cheng‐Kung University Tainan Taiwan; ^6^ Department of Electronic Engineering National Taipei University of Technology Taipei Taiwan; ^7^ Department of Surgery National Taiwan University Hospital Taipei Taiwan; ^8^ Graduate Institute of Brain & Mind Sciences College of Medicine National Taiwan University Taipei Taiwan; ^9^ Graduate Institute of Acupuncture Science College of Chinese Medicine China Medical University Taichung Taiwan

**Keywords:** endoscopic surgery, palmar hyperhidrosis, pulsed radiofrequency (PRF) stimulation, T2 sympathetic ganglion

## Abstract

**Objectives:**

Palmar hyperhidrosis (PH) exhibits excessive and unpredictable sweating. The most effective treatment for permanent cure is the ablation of thoracic sympathetic ganglia innervating hands. However, sympathectomy of T2 sympathetic ganglion by clipping or cauterization causes irreversible nerve damage, and results in a compensatory hyperhidrosis (CH). We herein used the pulsed radiofrequency (PRF) stimulation to reversibly block sympathetic ganglion to treat PH and avoid CH.

**Material and Methods:**

A bipolar electrode was implanted into the right T2 sympathetic trunk by endoscopic surgery and PRF was delivered through the electrode. The humidity (%) of right palm was measured to indicate sweating level.

**Results:**

Six out of 13 rats (46.2%) that received a 5‐min PRF stimulation on the T2 sympathetic trunk showed a decrease in the right palm humidity during the surgery. PRF stimulation significantly reduced humidity from 69.17% ± 0.72% obtained from baseline condition to 66.93% ± 0.69%. The humidity reduction was also observed at 10 min after the PRF stimulation. We further evaluated the effect of PRF stimulation 1 week after surgery and found that the PRF stimuli reduced right hand humidity in 5 out of 8 rats (62.5%). PRF stimulation significantly reduced humidity from 66.11% ± 0.81% obtained from sham operation control to 63.62% ± 0.82%. The percentage of right hand humidity obtained 10 min after PRF stimulation was also reduced to 63.38% ± 0.80%. Anesthetics have no effect on humidity.

**Conclusions:**

These results indicate that PRF stimulation of T2 sympathetic trunk reduces palm sweating in rats.

## 1 INTRODUCTION

Palmar hyperhidrosis (PH) exhibits excessive and unpredictable sweating, which mainly occurs on the palms and causes severe distress and discomfort in daily life. There are many surgical therapies, however, none of them is entirely satisfied in complications and adverse effects (Byrne, Walsh, & Hederman, 1990; Kopelman, Hashmonai, Ehrenreich, Bahous, & Assalia, 1996; Montessi et al., 2007). Nevertheless, transthoracic sympathetic intervention has been proven to achieve a long‐term relief of PH (Ambrogi et al., 2009; Baumgartner, 2008; Baumgartner, Bertin, & Konecny, 2009; Reisfeld & Berliner, 2008). Currently, the optimal therapy for treating PH is the transthoracic endoscopic sympathectomy (TES) (Baumgartner et al., 2009; Kao, 1992; Kao, Tsai, Lai, Hsiao, & Chiu, 1994). However, the major side effect of TES is compensatory hyperhidrosis (CH) on the trunk and thighs (Cetindag, Boley, Webb, & Hazelrigg, 2008). The CH is the greatest dissatisfaction of TES in treating PH (Dumont, 2008). The severity and mechanisms of CH caused by the denervation in sympathetic nerves remains unclear (Katara et al., 2007; Kopelman & Hashmonai, 2008; Miller & Force, 2007). Therefore, avoiding the complication of CH becomes an essential goal in the treatment of PH. There is no any surgical therapy has been successfully resolving this problem.

In the past, sympathecotomy was mainly performed by cutting or clipping T2 ganglia (Chuang & Liu, 2002; Hsia, Chen, Hsu, Shai, & Yang, 1999; Kim et al., 2001; Lin, Kuo, & Chou, 2002; Lin, Mo, Lee, Ng, & Hwang, 1998). The occurrence rate of CH is up to 86% and a considerable number of patients had to undergo resympathecotomy for the recurrent hyperhidrosis (Lin, 2001; Lin et al., 2002). Disruption of the sympathetic ganglia by clipping or cauterization can effectively relieve the PH, however, the adverse effect of CH is difficult to compromise because the sympathetic function of T2 has been irreversibly blocked. Recent studies reveal that the sympathetic function is irreversibly interrupted when clipping of T2 ganglia exhibits efficiency to relieve PH. The Wallerian degeneration and the loss of neuronal axons will be occurred within 10 days after clipping. Absence of myelinated and unmyelinated fibers following the removal of the T2 ganglion clip suggests that no nerve regenerates afterwards (Candas et al., 2012; Loscertales et al., 2012).

The PRF neurostimulation has been demonstrated to reversibly block the sensory afferents for pain transmission (Carles et al., 2001; Dupré, 1992). It has also been shown that the PRF stimulation of sympathetic nerves relieves pain (Sellgren, Ponten, & Wallin, 1990). Based upon these observations, in this study we tried to elucidate the efficacy of reducing sweating in rats by applying a reversible sympathetic blockade with the PRF stimuli on the T2 sympathetic ganglion.

## 2 METHODS

### 2.1 Animals

Male Sprague‐Dawley rats with 400–650 g were used in this study. Rats were obtained from National Laboratory Animal Breeding and Research Center, Taiwan. All animals were separately housed in a recording cage and the light‐dark cycle is controlled in a 12:12 hr light/dark (L:D) cycle. The temperature was maintained at 23 ± 1°C, and food and water were provided ad libitum. All of the following procedures have been approved by the Institutional Animal Care and Use Committee (IACUC) of National Taiwan University.

### 2.2 Transthoracic endoscopic surgery and implantation of bipolar electrode

**Figure 1:**
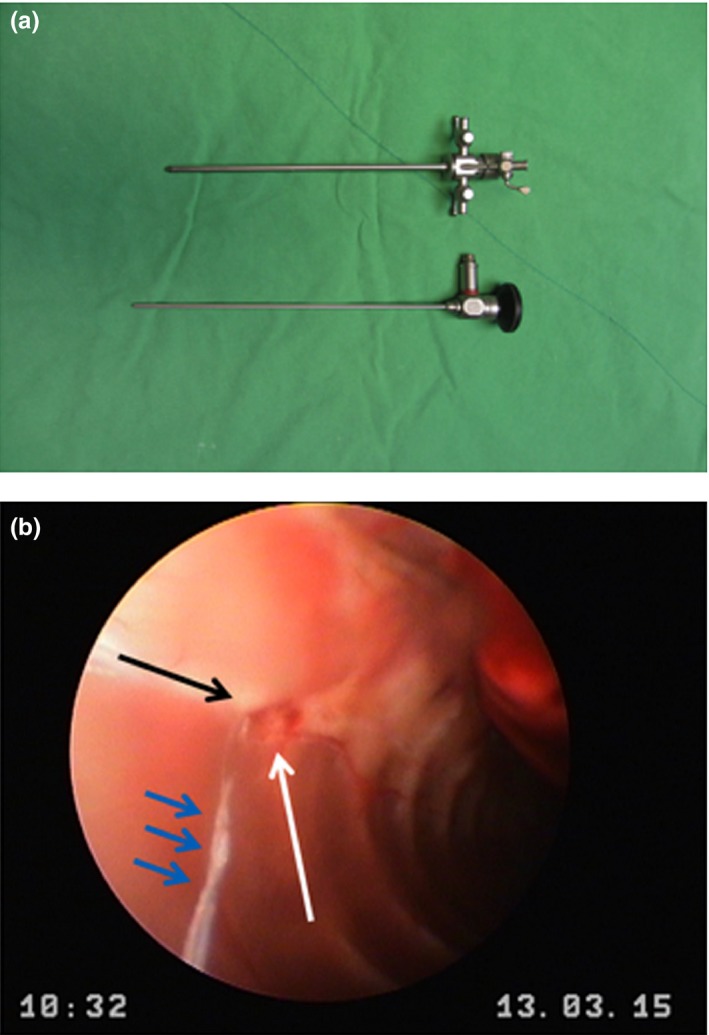
(a) The rhinoscopy (64019BA 30° 2.7 mm Rhinoscopy, KARL STORZ GmbH & Co, Germany) was used to approach to and fix the electrodes adjacent to the thoracic sympathetic trunk. (b) Endoscopic image. The black arrow indicates the sympathetic trunk, and white arrow refers to T2 rib. The blue arrows represent the implanted bipolar electrodes

Atropine (0.1 mg/kg, subcutaneously injection), Meloxican (1 mg/kg, intramuscular injection), and Midazolam (0.67 mg/kg, intramuscular injection) were administrated to rats as pre‐anesthesia medications. Atropine was used to prevent salivation and Meloxican was used to reduce pain. Midazolam was given to sedate subjects. Rats were put into an anesthetic chamber with Isoflurane delivering with the rate of 5% in the oxygen flow to induce anesthesia. Rats were then intubated with an 18‐gauge catheter. Isoflurane was delivered with the rate of 1.5%~3% for maintenance of anesthesia, and it was reduced to 1%~2% when the palmar humidity was recorded (see the following protocol). Thoracic endoscopic surgery was performed to implant a bipolar electrode to the right T2 sympathetic ganglion. The rat was placed in a left recumbent position. A pre‐heated water bag was placed beneath the rat in order to prevent hypothermia. An incision was made at the lateral T2‐T3 intercostal area. We used a mosquito forcep to carefully separate muscles and to approach the thoracic cavity. A rhinoscopy (64019BA 30° 2.7 mm Rhinoscopy, KARL STORZ GmbH & Co, Germany, Figure 1) was used to fix the electrodes to the T2 sympathetic ganglion. The bipolar electrode was made by passing 2 electrical wires (model # M148340, California fine wire company, Grover beach, CA, USA) into the cavity of a 27‐gauge needle. The vertical mattress stitch was placed to suture the incision. Oxygen was provided throughout the recovery period until the rat was awake. The sutured site was anointed with a Sindine ointment.

### 2.3 Humidity recording

A Humidity and Temperature Sensor IC (SHT1x, Sensirion Switzerland Co. Ltd) was used to measure the humidity of four palms in rats. The sensor was connected to a capsule to measure the palmar humidity by tightly covering the palm. The humidity was recorded every 5 s and for a total of 300 s in each recording time. The recordings of palmar humidity were duplicated for each time of recording. We acquired the humidity before the PRF stimuli and at 10 min after the PRF stimuli during the surgery protocol and at 1 week after the surgery. Since we determined the humidity generated by the palm inside the capsule, the humidity would be increased gradually and then saturated. The humidity maintains consistently with the environmental humidity (approximately around 42% humidity), when the capsule is covered on the bench at room temperature. This difference indicates that the recording of palmar humidity is a specific measurement rather than an artificial result.

### 2.4 Pulsed radiofrequency (PRF) generator

**Figure 2:**
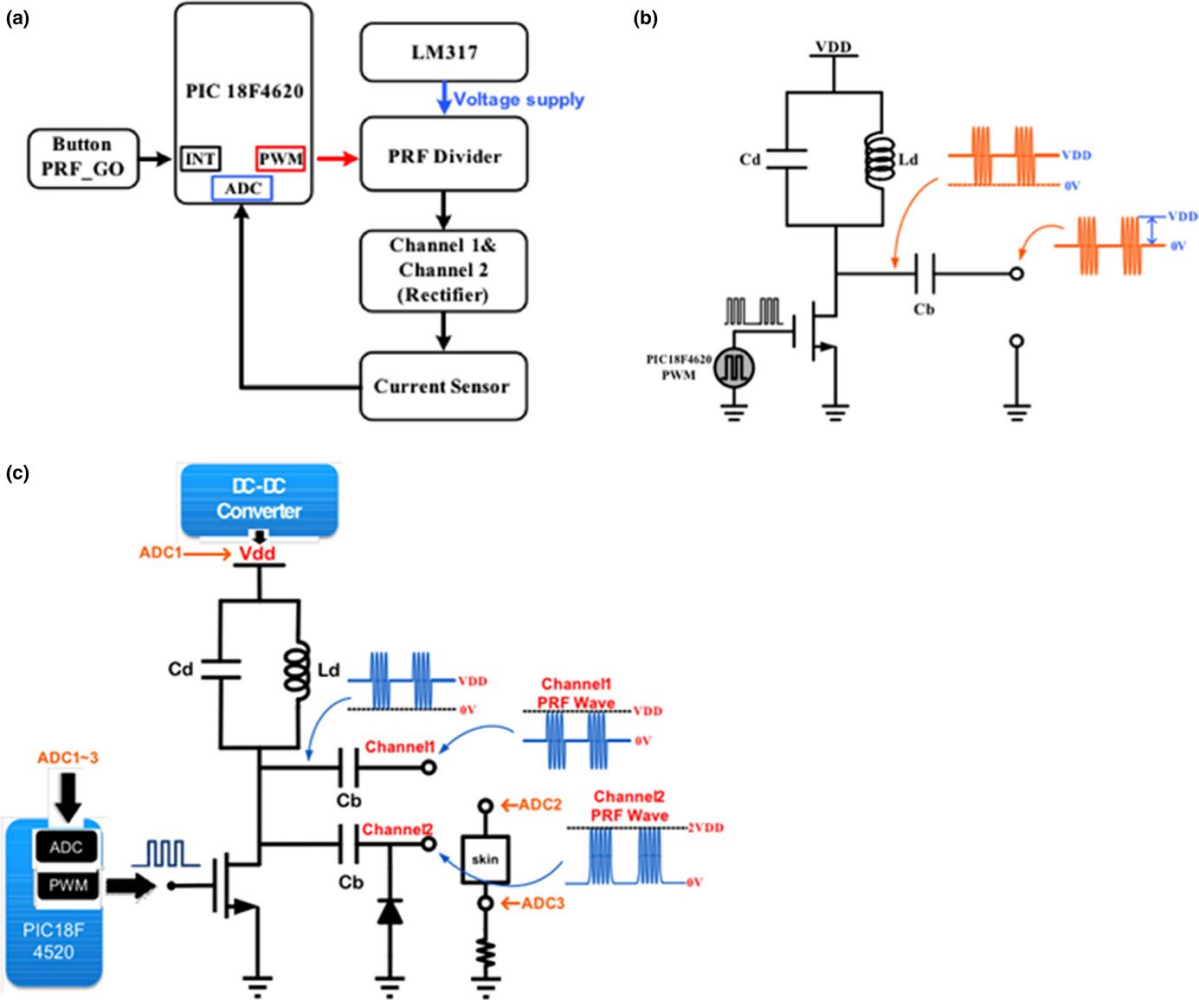
(a) Pulsed radiofrequency (PRF) generator architecture. PRF is generated through the PIC18F4620 PWM signal to drive the PRF Driver. After PWM starting, PRF Driver output wave would pass through two channels, channel 1 and channel 2. Channel 1 function filter out superfluous DC value and have rectification function at channel 2. (b) PRF divider circuit. The switch signal of MOSFET was supplied by PWM of PIC18F4620 modulation. Where Ld and Cd composition by the resonant cavity, resonance frequency should be the same with MOS switching frequency. (c) Traditional and rectified PRF waveform circuit. Two waveforms were designed: the first waveform, traditional PRF in Channel 1, was simply without direct spin wave; the second waveform rectified PRF

The PRF was generated through the PIC18F4620 pulse width modulation (PWM) signal to drive the PRF Driver (Figure 2a). The PWM was determined when a button of PRF_GO was turned on. We designed a Boost Converter circuit as a power source to provide a high DC voltage to the PRF driver. The DC voltage value was decided by a variable resistor and the voltage range is between 10 V and 100 V. After PWM started, the output wave of PRF Driver could pass through two channels, the channel 1 and channel 2. The function of channel 1 filtered out the superfluous DC value, and subsequently rectified the channel 2.

In Figure 2b, the switch signal of the metal–oxide–semiconductor field‐effect transistor (MOSFET) was supplied by PWM of PIC18F4620 modulation. Where Ld and Cd were composed by the resonant cavity, the resonance frequency should be the same as the MOS switching frequency. Resonance frequency formula is as follows: ƒ = $\frac{1}{2\pi \sqrt{LC}}$. The Ld and Cd values must be loaded. But, when they were loaded with high values, the waveform would be distorted. Therefore, the Ld and Cd must be with a high quality factor (Q). Parallel Q value formula is as follows: Q = $R\sqrt{C/L}$. According to the formula, if the Q value is higher, the Ld value needs to be lower. However, the Ld value could not be lower, because the decline of Ld values could lead the burden of Boost Conventer to be heavy. Therefore, Ld and Cd must strike a balance with the Boost Converter. This not only causes a minor waveform distortion, but also provides a high voltage by the Boost Converter. When the PWM started, the PRF driver produced the DC component of a sine wave that was the same frequency as PWM and the amplitude was 0~2 VDD. Then, A capacitance, Cb excluded the DC component of a sine wave. We obtained a traditional PRF waveform.

In the PRF driver we designed two waveforms: the first waveform, a traditional PRF in the Channel 1 (Figure 2c), was simply without the direct spin wave; the second waveform was a rectified PRF (Figure 2b). PRF was rectified because of more diodes in the circuit of Channel 2. The diodes clamped the original PRF waveform and the position of the center of clamp VDD, thus formed twice VDD to a 0 volt waveform. There will be a loss in the actual circuit and therefore the peak‐to‐peak voltage of the Channel 1 and Channel 2 was not equal to the twice of VDD. VDD was about eighty percent to ninety percent twice, when the input voltage is higher than the more obvious loss of voltage.

### 2.5 Experimental protocols

**Figure 3:**
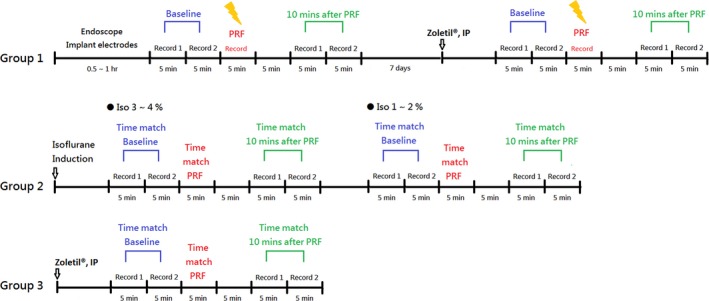
The experimental protocols. Three groups of animals were used in this study

The experimental protocols are depicted in Figure 3. Rats were divided into three groups. Rats of the first group (*n* = 13) immediately received the PRF stimuli after the endoscopic surgery and electrode implantation, and also received the PRF stimuli 1 week after recovery from the surgery. Rats were anesthetized by 1%~2% of Isoflurane when they received the PRF stimulation during the endoscopic surgery. Rats were anesthetized by Zoletil (20 mg/kg, intraperitoneal (IP) injection) when they received the PRF stimulation 1 week after recovery from the surgery. The baseline of palmar humidity was recorded twice before the PRF stimuli during the surgery and at 1 week after recovery, which served as the sham control for the operative effect. The second group (*n* = 4) was used as a control to assess the anesthetic effect of Isoflurane during the endoscopic surgery on palmar humidity. The third group (*n* = 5) received Zoletil by IP injection only as a control to compare with that after the postsurgery PRF stimulation. The cardiovascular parameters and the blood oxygen saturation levels (SpO_2_) were monitored during all of the experimental conditions. However, there was no any significantly statistical alteration, suggesting that the electrode implantation and PRF stimulation may not have cardiovascular adverse effects.

### 2.6 Statistical analysis

The values of humidity (%) were present as mean ± *SEM* %. The statistical analyses were performed by SAS‐9.3 software. The comparison between values obtained from different groups was analyzed by the repeated measures ANOVA. If statistically significant differences were detected, post hoc (Duncan's) multiple range tests were used. If the *p* value is <.05, then it was taken as indicators of statistically significant difference.

## 3 RESULTS

### 3.1 The effects of RPF stimulation on right palmar humidity during endoscopic surgery

**Figure 4:**
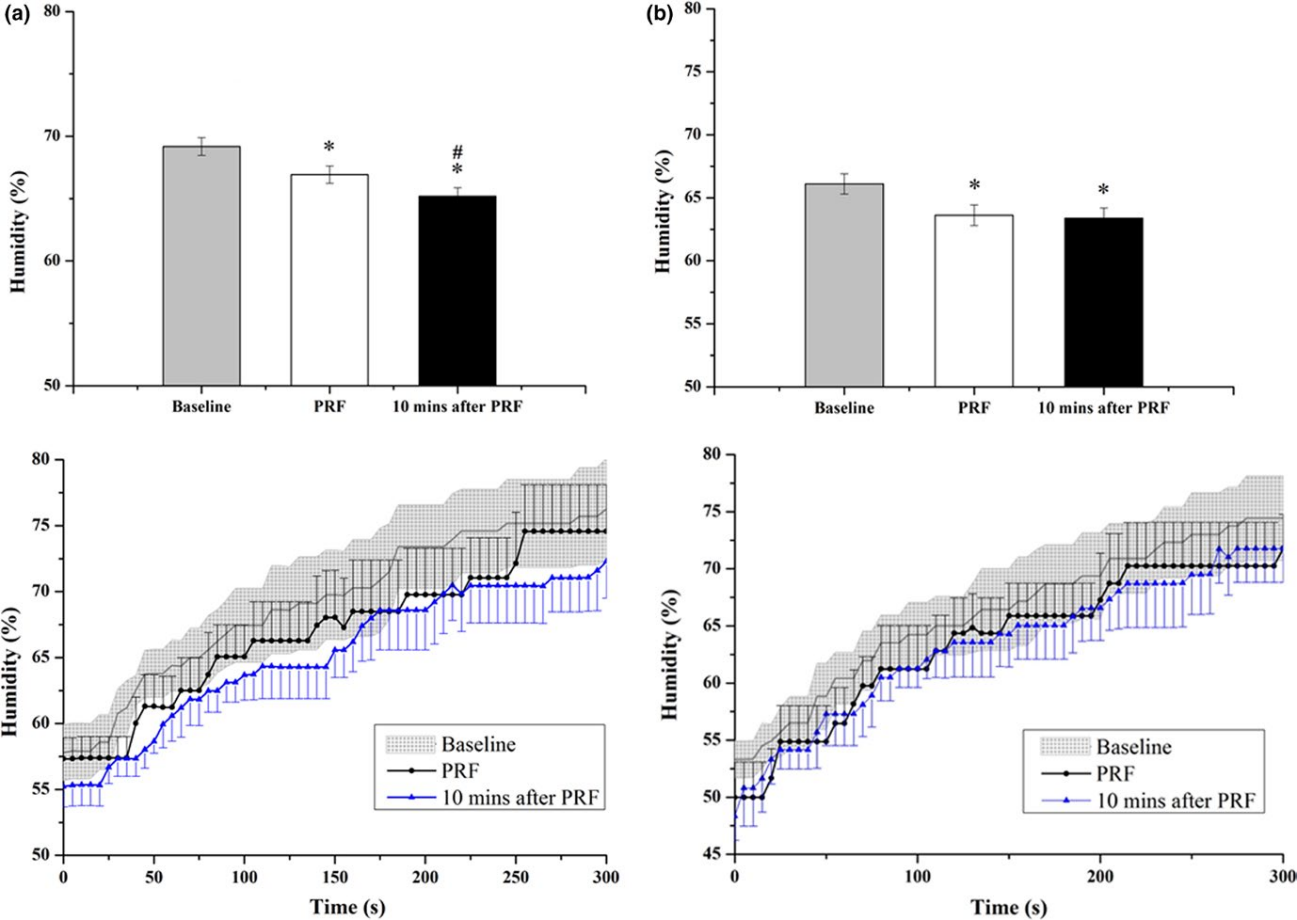
(a) The percentage of right palm humidity obtained from successful group during surgery (*n* = 6). PRF stimulation significantly decreased the humidity of right palm, and also diminished it 10 min after PRF stimulation. * refers to a statistically significant difference comparing with the baseline, and # refers to a statistically significant difference comparing with the PRF period. (b) The percentage of right palm humidity obtained 1 week after surgery (*n* = 5). The PRF stimulation statistically inhibited the sweating of right palm. The humidity obtained 10 min after PRF was also decreased. * refers to a statistically significant difference comparing with the baseline

In group 1, six out of thirteen rats that received the PRF stimulation on the right T2 ganglion following endoscopic surgery immediately showed a decrease in the right palm humidity (with 46.2% of successful rate), when comparing to the baseline (control) recording in which the bipolar stimulating electrode was implanted but without stimuli. The percentage of right palmar humidity acquired from the baseline recording was 69.17% ± 0.72% (*n* = 6; Figure 4a). The PRF stimulation statistically decreased the right palmar humidity either during the stimulation or 10 min after the PRF stimulation. The percentages of right palmar humidity were 66.93% ± 0.69% (*n* = 6; *p* < .05 vs. baseline (control) recording, *F* = 36.66, repeated measures ANOVA) and 65.20% ± 0.67% (*n* = 6; *p* < .05 vs. baseline (control) recording, *F* = 36.66, repeated measures ANOVA) during the PRF stimuli and 10 min after PRF stimulation, respectively, (Figure 4a). Both humidity percentages from the left palmar and the right and left hindlimbs acquired after the PRF stimulation of right T2 ganglion did not differ from the control (data not shown), indicating the specificity of PRF stimulation on the right T2 ganglion.

### 3.2 The effects of PRF stimulation on right palmar humidity one week after recovery from surgery

Our result has demonstrated there was no difference on the right palmar humidity when comparing the change between naïve rats and the rats implanted the bipolar electrode without the PRF stimulation (a sham control for operation; data not shown). In group 1, we performed the PRF stimuli again 1 week after recovery from the endoscopic surgery. The PRF stimulation successfully suppressed the right palmar humidity in 5 out of 8 rats (with 62.5% of successful rate). The baseline recording (the sham control for operation) of right palmar humidity was 66.11% ± 0.81% (*n* = 5; Figure 4b). The PRF stimulation decreased the percentage of right palmar humidity to 63.62% ± 0.82% (*n* = 5, *p* < .05 vs. baseline, *F* = 14.55, repeated measures ANOVA, Figure 4b). The percentage of right palmar humidity required from 10 min after the PRF stimulation was also significantly decreased to 63.38% ± 0.80% (*n* = 5, *p* < .05 vs. baseline, *F* = 14.55, repeated measures ANOVA, Figure 4b). Both percentages of humidity from the left palm and the right and left hindlimbs acquired after the PRF stimulation did not differ from the control (data not shown).

### 3.3 The effects of Isoflurane on the right palmar humidity

**Figure 5:**
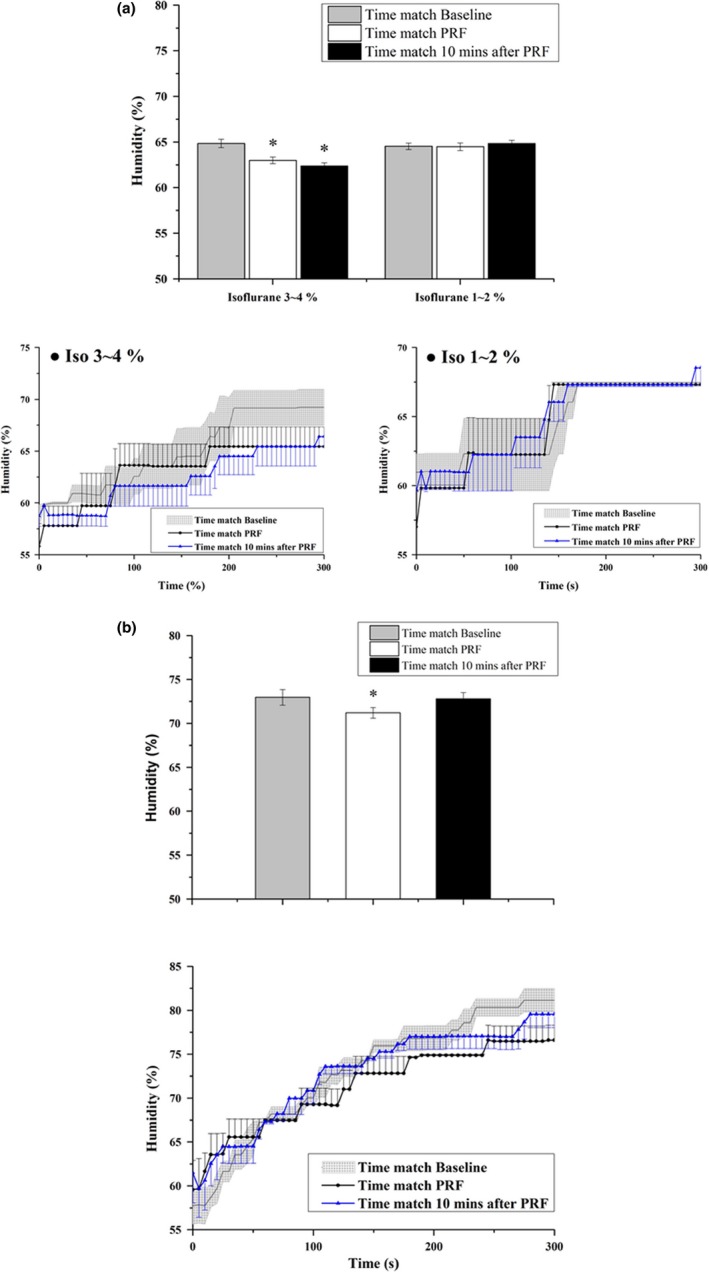
(a) The percentage of right palm humidity obtained from rats receiving Isoflurane maintenance. Isoflurane delivered at the rate of 3%~4% decreased the humidity of right palm during the time match PRF and 10 min after PRF, while Isoflurane delivered at the rate of 1%~2% showed no significant influence on either time windows. * refers to a statistically significant difference comparing with the time match baseline. (b) The percentage of right palm humidity obtained from rats receiving Zoletil^®^ injection. Zoletil^®^ significantly decreased the humidity of right palm obtained during the time match PRF. The humidity of right palm obtained during the time match baseline and 10 min after PRF showed no significant alteration. * refers to a statistically significant difference comparing with the time match baseline

The humidity of right palm was acquired while delivering either high or low concentration of Isoflurane in the oxygen flow to maintain anesthesia. With delivering Isoflurane at the rate of 3%~4% in oxygen flow, the percentage of right hand humidity obtained from the time matching baseline recording was 64.84% ± 0.46%, and that required from the time matching the PRF stimulation and 10 min after PRF stimulation declined to 62.98% ± 0.37% (*n* = 4, *p* < .05 vs. baseline, *F* = 27.72, repeated measures ANOVA) and 62.39% ± 0.33% (*n* = 4, *p* < .05 vs. baseline, *F* = 27.72, repeated measures ANOVA), respectively, (Figure 5a). With delivering Isoflurane at the rate of 1%~2% in oxygen flow, the right palm humidity of the time matching the baseline recording, the PRF stimulation, and 10 min after PRF stimulation showed no significant difference (Figure 5a). The percentage of right palm humidity obtained from the time match baseline, PRF stimulation, and 10 min after PRF stimulation was 64.52% ± 0.35% (*n* = 3, *p* > .05, repeated measures ANOVA), 64.47% ± 0.41% (*n* = 3, *p* > .05, repeated measures ANOVA) and 64.83% ± 0.36% (*n* = 3, *p* > .05, repeated measures ANOVA), respectively. This result suggests that the effect of PRF on humidity suppression during the endoscopic surgery was not influenced by the anesthetic of 1%~2% Isoflurane.

### 3.4 The effects of Zoletil on the right palmar humidity

The humidity of right palm obtained from the time matching the PRF stimulation after receiving Zoletil was significantly decreased comparing to that obtained from the time matching baseline recording (*n* = 5, *p* < .05, *F* = 6.90, repeated measures ANOVA; Figure 5b). The humidity of right palm obtained from the time matching 10 min after PRF stimulation in rats received Zoletil showed no statistical difference from that obtained from the time matching baseline. The percentage of right palm humidity obtained from the time matching the baseline, the PRF stimulation, and 10 min after PRF stimulation were 72.39% ± 0.96%, 70.56% ± 0.72%, and 72.41% ± 0.81%, respectively. This result suggests that Zoletil did not influence the humidity suppression at 10 min after PRF. The β value was 0.19 and the power was 0.81 when comparing three control groups, the naïve group, Isoflurane group and Zoletil group. This result suggests there was no type II statistical error, although the sample size was small.

## 4 DISCUSSION

There are many options available for sympathetic chain interruption, including the sympathectomy, the clipping of the sympathetic chain and the ramicotomy achieve permanent disruption. However, the common complication of these procedures is the CH (Lin et al., 1998). The complication of CH may vary in degree. The mild degree complication reportedly affects from 15% to 90% of patients, and the percentage of severe degree of CH is between 1% and 30% (Lyra Rde et al., 2008).

Some authors who recommend the clipping of sympathetic chain have reported an acceptable outcome following unclipping, in which the improvement in symptoms is ranging from 52% to 100% (Lin & Chou, 2004; Reisfeld, Nguyen, & Pnini, 2002). However, literatures indicated a failure of reversibility, which is more likely related to perineural damage of the nerve by clipping (Kang et al., 2008; Loscertales et al., 2012; Zaretsky & Shofti, 2007). Although Kang et al. suggest that the improvement in compensatory sweating is obtained 4 weeks after unclipping (Loscertales et al., 2012), Loscertales et al. found the Wallerian degeneration and axon loss at 10 days after clip removal and there is no evidence of nerve regeneration (Loscertales et al., 2012). The almost total absence of myelinated and unmyelinated fibers following the clip removal suggests no nerve regeneration (Zaretsky & Shofti, 2007). Therefore, once the clipping is effective enough to relieve the PH, it is no more considered to be a reversible interruption in the sympathectic function (Zaretsky & Shofti, 2007). Because of the complication of CH and the nerve degeneration, a reversible blockade of sympathetic activity seems become an optimal option for PH treatment.

We designed a neurostimulator based on PRF as a tool to produce a reversibly sympathetic blockade. The idea of current design is to let patients control the stimulation time to reduce palm sweating as demand. In the majority of existing studies, the neurostimulation was applied on the peripheral nerves (axons). However, the sympathetic ganglion could serve as a feasible target site for the neurostimulation, since it contains not only nerve fibers but also nerve somas. Furthermore, it has been established in previous studies that various animals respond differently to the activation of the tested stimulator (Kopelman, Costa, Bejar, Zaretsky, & Hashmonai, 2012). Although the endoscopic surgery was conducted delicately and the endoscope was approached to the T2 sympathetic ganglion as precisely as possible, in fact, an accidental injury on the sympathetic ganglion was inevitable during the manipulation, which may further result in edema as described by Kopelman et al. (Samuelsson, Claes, & Drott, 1994). However, these pathological changes by endoscopic surgery would gradually disappear, and finally there is no any irreversible damage of the ganglion. The inflammatory process that accompanied the pathological changes might cause the palmar sweating. In order to clearly determine the effect of PRF stimulation on the palmar sweating, we repeated the PRF stimulation 1 week after recovery from the endoscopic surgery. We delivered a repeat PRF stimulation and recorded the palmar sweating twice. We found that the palmar sweating was reduced in 5 out of 8 rats and reached a successful rate of 62.5%. This result explicitly indicates that an accidental injury on ganglion cells could recover in the period of 1 week after surgery, and the PRF stimulation effectively reduced palmar hyperhidrosis as shown in Figure 4b.

Horner's syndrome is caused by the deficiency of sympathetic activity. The major signs and symptoms of Horner's syndrome include miosis, ptosis, and anhidrosis (Ropper & Brown, 2005). The occurrence of Horner's syndrome is common with patients who receive the sympathectomy. However, our previous studies indicate that the Horner's syndrome is not encountered in our patients who received the T2 sympathectomy, because sympathetic fibers related to Horner's syndrome come from the stellate ganglion or from a higher level, located at or above the dome of the thoracic cavity (Kao, 1992; Kao et al., 1994). Both heart rate and blood pressure were not changed in our previous studies during and after the sympathectomy (Kao, 1992; Kao et al., 1994). In our current study, we did not observe any signs of deficiency in the sympathetic activity, including the alteration in blood pressure and heart rate during and after the PRF stimulation in rats (data not shown). This observation indicated that the sympathetic tone was minimally altered by the PRF stimulation at the T2 ganglion.

Theoretically, Isoflurane, a general anesthetic, could induce vasodilatation and consequently affect sweating by inhibiting the sympathetic system as described in others experiments (Sellgren, Ejnell, Elam, Ponten, & Wallin, 1994; Sellgren et al., 1990; Stevens et al., 1971). In this study, the palmar sweating was not influenced (Group 2 data) by 1%~2% Isoflurane; however, the palmar sweating was decreased by 3%–4% Isoflurane anesthesia. When we determined the humidity degree of palmar sweating by application of the PRF stimulation during the endoscopic surgery, the level of Isoflurane was reduced to 1%~2%. Therefore, the effect of reduction in palmar sweating by the PRF during the endoscopic surgery may not be interfered by Isoflurane. Our findings also suggested that the general anesthetic Isoflurane inhibiting the sympathetic function depends upon the concentrations of the Isoflurane. We also needed to measure the sympathetic inhibition of another general anesthetics, Zoletil, by the IP injection used in the repeated PRF experiment 1 week after the endoscopic surgery. We tested whether the palmar sweating is altered by Zoletil, and found that Zoletil decreased palmar sweating during the time matching the PRF stimulation, but Zoletil had no effect on palmar humidity during the time matching the 10 min after the PRF stimulation. Therefore, we confirmed that the reduction in palm humidity 10 min after the PRF stimulation is the effect of PRF stimulation rather than the effect of Zoletil.

## 5 CONCLUSION

In conclusion, this study indicated that application of PRF stimulation with implantation of a miniature stimulator and an adequate the stimulation protocol on the thoracic sympathetic trunk can achieve a reversible sympathetic blockade to reduce palmar sweating. Based on this study, we believe that applying the newly designed PRF stimulation at the T2 sympathetic ganglion could relieve PH and this could avoid the distressing complication of CH, since the sympathetic blockade is reversible. The further study will try to make the PRF stimulation fabricated into a wireless micro‐chip for a long‐termed use to relieve the palmar hyperhidrosis.

## DISCLOSURE

Authors have indicated no financial conflicts of interest.

## AUTHOR CONTRIBUTIONS

Conception and design: FC Chang, ML Lin, MC Kao, HW Chiu, SC Lin. Acquisition of data: TR Huang. Analysis and interpretation of data: TR Huang, FC Chang. Drafting the article: TR Huang, ML Lin. Critically revising the article: FC Chang, MC Kao. Approved the final version of the manuscript on behalf of all authors: FC Chang. Statistical analysis: TZ Huang. Study supervision: FC Chang.

## References

[brb3833-bib-0001] Ambrogi, V. , Campione, E. , Mineo, D. , Paterno, E. J. , Pompeo, E. , & Mineo, T. C. (2009). Bilateral thoracoscopic T2 to T3 sympathectomy versus botulinum injection in palmar hyperhidrosis. Annals of Thoracic Surgery, 88, 238–245. 19559233 10.1016/j.athoracsur.2009.04.003

[brb3833-bib-0002] Baumgartner, F. J. (2008). Surgical approaches and techniques in the management of severe hyperhidrosis. Thoracic Surgery Clinics, 18, 167–181. 18557590 10.1016/j.thorsurg.2008.01.005

[brb3833-bib-0003] Baumgartner, F. J. , Bertin, S. , & Konecny, J. (2009). Superiority of thoracoscopic sympathectomy over medical management for the palmoplantar subset of severe hyperhidrosis. Annals of Vascular Surgery, 23, 1–7. 18619780 10.1016/j.avsg.2008.04.014

[brb3833-bib-0004] Byrne, J. , Walsh, T. N. , & Hederman, W. P. (1990). Endoscopic transthoracic electrocautery of the sympathetic chain for palmar and axillary hyperhidrosis. British Journal of Surgery, 77, 1046–1049. 2131796 10.1002/bjs.1800770931

[brb3833-bib-0005] Candas, F. , Gorur, R. , Haholu, A. , Yiyit, N. , Yildizhan, A. , Gezer, S. , … Isitmangil, T. (2012). The effects of clipping on thoracic sympathetic nerve in rabbits: Early and late histopathological findings. The Thoracic and Cardiovascular Surgeon, 60, 280–284. 22411756 10.1055/s-0031-1299573

[brb3833-bib-0006] Carles, M. , Pulcini, A. , Macchi, P. , Duflos, P. , Raucoules‐Aime, M. , & Grimaud, D. (2001). An evaluation of the brachial plexus block at the humeral canal using a neurostimulator (1417 patients): The efficacy, safety, and predictive criteria of failure. Anesthesia and Analgesia, 92, 194–198. 11133626 10.1097/00000539-200101000-00037

[brb3833-bib-0007] Cetindag, I. B. , Boley, T. M. , Webb, K. N. , & Hazelrigg, S. R. (2008). Long‐term results and quality‐of‐life measures in the management of hyperhidrosis. Thoracic Surgery Clinics, 18, 217–222. 18557594 10.1016/j.thorsurg.2008.01.009

[brb3833-bib-0008] Chuang, K. S. , & Liu, J. C. (2002). Long‐term assessment of percutaneous stereotactic thermocoagulation of upper thoracic ganglionectomy and sympathectomy for palmar and craniofacial hyperhidrosis in 1742 cases. Neurosurgery, 51, 963–970. 12234404 10.1097/00006123-200210000-00021

[brb3833-bib-0009] Dumont, P. (2008). Side effects and complications of surgery for hyperhidrosis. Thoracic Surgery Clinics, 18, 193–207. 18557592 10.1016/j.thorsurg.2008.01.007

[brb3833-bib-0010] Dupré, L. J. (1992). The neurostimulator in loco‐regional anesthesia. Cahiers d'Anesthesiologie, 40, 503–510. 1477773

[brb3833-bib-0011] Hsia, J. Y. , Chen, C. Y. , Hsu, C. P. , Shai, S. E. , & Yang, S. S. (1999). Outpatient thoracoscopic limited sympathectomy for hyperhidrosis palmaris. Annals of Thoracic Surgery, 67, 258–259. 10086571 10.1016/s0003-4975(98)01208-9

[brb3833-bib-0012] Kang, C. W. , Choi, S. Y. , Moon, S. W. , Cho, D. G. , Kwon, J. B. , Sim, S. B. , … Jo, K. H. (2008). Short‐term and intermediate‐term results after unclipping: What happened to primary hyperhidrosis and truncal reflex sweating after unclipping in patients who underwent endoscopic thoracic sympathetic clamping? Surgical Laparoscopy Endoscopy & Percutaneous Techniques, 18, 469–473. 10.1097/SLE.0b013e31817e91f8 18936668

[brb3833-bib-0013] Kao, M. C. (1992). Video endoscopic sympathectomy using a fiberoptic CO2 laser to treat palmar hyperhidrosis. Neurosurgery, 30, 131–135. 1738444 10.1227/00006123-199201000-00026

[brb3833-bib-0014] Kao, M. C. , Tsai, J. C. , Lai, D. M. , Hsiao, Y. Y. , & Chiu, M. J. (1994). Autonomic activities in hyperhidrosis patients before, during, and after endoscopic laser sympathectomy. Neurosurgery, 34, 262–268. 8177387 10.1227/00006123-199402000-00009

[brb3833-bib-0015] Katara, A. N. , Domino, J. P. , Cheah, W. K. , So, J. B. , Ning, C. , & Lomanto, D. (2007). Comparing T2 and T2‐T3 ablation in thoracoscopic sympathectomy for palmar hyperhidrosis: A randomized control trial. Surgical Endoscopy, 21, 1768–1771. 17404794 10.1007/s00464-007-9241-9

[brb3833-bib-0016] Kim, B. J. , Oh, B. S. , Park, Y. K. , Jang, W. C. , Suh, H. J. , & Im, Y. H. (2001). Microinvasive video‐assisted thoracoscopic sympathicotomy for primary palmar hyperhidrosis. American Journal of Surgery, 181, 540–542. 11513781 10.1016/s0002-9610(01)00627-4

[brb3833-bib-0017] Kopelman, D. , Costa, M. G. , Bejar, J. , Zaretsky, A. , & Hashmonai, M. (2012). Attempted reversible sympathetic ganglion block by an implantable neurostimulator. Interactive Cardiovascular and Thoracic Surgery, 14, 605–609. 22316522 10.1093/icvts/ivr137 PMC3329309

[brb3833-bib-0018] Kopelman, D. , & Hashmonai, M. (2008). The correlation between the method of sympathetic ablation for palmar hyperhidrosis and the occurrence of compensatory hyperhidrosis: A review. World Journal of Surgery, 32, 2343–2356. 18797962 10.1007/s00268-008-9716-4

[brb3833-bib-0019] Kopelman, D. , Hashmonai, M. , Ehrenreich, M. , Bahous, H. , & Assalia, A. (1996). Upper dorsal thoracoscopic sympathectomy for palmar hyperhidrosis: Improved intermediate‐term results. Journal of Vascular Surgery, 24, 194–199. 8752028 10.1016/s0741-5214(96)70093-9

[brb3833-bib-0020] Lin, T. S. (2001). Video‐assisted thoracoscopic “resympathicotomy” for palmar hyperhidrosis: Analysis of 42 cases. Annals of Thoracic Surgery, 72, 895–898. 11565677 10.1016/s0003-4975(01)02852-1

[brb3833-bib-0021] Lin, T. S. , & Chou, M. C. (2004). Treatment of palmar hyperhidrosis using needlescopic T2 sympathetic block by clipping: Analysis of 102 cases. International Surgery, 89, 198–201. 15730099

[brb3833-bib-0022] Lin, T. S. , Kuo, S. J. , & Chou, M. C. (2002). Uniportal endoscopic thoracic sympathectomy for treatment of palmar and axillary hyperhidrosis: Analysis of 2000 cases. Neurosurgery, 51(suppl 5), S84–S87. 12234434

[brb3833-bib-0023] Lin, C. C. , Mo, L. R. , Lee, L. S. , Ng, S. M. , & Hwang, M. H. (1998). Thoracoscopic T2‐sympathetic block by clipping—A better and reversible operation for treatment of hyperhidrosis palmaris: Experience with 326 cases. European Journal of Surgery. Supplement 580, 13–16. 10.1080/11024159850191067 9641378

[brb3833-bib-0024] Loscertales, J. , Congregado, M. , Jimenez‐Merchan, R. , Gallardo, G. , Trivino, A. , Moreno, S. , … Galera‐Ruiz, H. (2012). Sympathetic chain clipping for hyperhidrosis is not a reversible procedure. Surgical Endoscopy, 26, 1258–1263. 22083329 10.1007/s00464-011-2023-4

[brb3833-bib-0025] Lyra Rde, M. , Campos, J. R. , Kang, D. W. , Loureiro Mde, P. , Furian, M. B. , Costa, M. G. , … Sociedade Brasileira de Cirurgia Torácica . (2008). Guidelines for the prevention, diagnosis and treatment of compensatory hyperhidrosis. Jornal Brasileiro de Pneumologia, 34, 967–977. 19099105 10.1590/s1806-37132008001100013

[brb3833-bib-0026] Miller, D. L. , & Force, S. D. (2007). Outpatient microthoracoscopic sympathectomy for palmar hyperhidrosis. Annals of Thoracic Surgery, 83, 1850–1853. 17462412 10.1016/j.athoracsur.2006.11.030

[brb3833-bib-0027] Montessi, J. , Almeida, E. P. , Vieira, J. P. , Abreu Mda, M. , Souza, R. L. , & Montessi, O. V. (2007). Video‐assisted thoracic sympathectomy in the treatment of primary hyperhidrosis: A retrospective study of 521 cases comparing different levels of ablation. Jornal Brasileiro de Pneumologia, 33, 248–254. 17906784 10.1590/s1806-37132007000300004

[brb3833-bib-0028] Reisfeld, R. , & Berliner, K. I. (2008). Evidence‐based review of the nonsurgical management of hyperhidrosis. Thoracic Surgery Clinics, 18, 157–166. 18557589 10.1016/j.thorsurg.2008.01.004

[brb3833-bib-0029] Reisfeld, R. , Nguyen, R. , & Pnini, A. (2002). Endoscopic thoracic sympathectomy for hyperhidrosis: Experience with both cauterization and clamping methods. Surgical Laparoscopy Endoscopy & Percutaneous Techniques, 12, 255–267. 10.1097/00129689-200208000-00011 12193821

[brb3833-bib-0030] Ropper, A. H. , & Brown, R. H. (2005). Disorders of ocular movement and pupillary function In Ropper A. H., & Brown R. H. (Eds.), Adams and Victor's Principles of Neurology, 8^th^ edn (pp. 222–245). New York, NY: McGraw‐Hill Professional.

[brb3833-bib-0031] Samuelsson, H. , Claes, G. , & Drott, C. (1994). Endoscopic electrocautery of the upper thoracic sympathetic chain: A safe and simple technique for treatment of sympathetically maintained pain. European Journal of Surgery. Supplement 572, 55–57. 7524786

[brb3833-bib-0032] Sellgren, J. , Ejnell, H. , Elam, M. , Ponten, J. , & Wallin, B. G. (1994). Sympathetic muscle nerve activity, peripheral blood flows and baroreceptor reflexes in humans during propofol anesthesia and surgery. Anesthesiology, 80, 534–544. 8141450 10.1097/00000542-199403000-00009

[brb3833-bib-0033] Sellgren, J. , Ponten, J. , & Wallin, B. G. (1990). Percutaneous recording of muscle nerve sympathetic activity during propofol nitrous oxide and isoflurane anesthesia in humans. Anesthesiology, 73, 20–27. 2360736 10.1097/00000542-199007000-00004

[brb3833-bib-0034] Stevens, W. C. , Cromwell, T. H. , Halsey, M. J. , Eger, E. I. , Shakespeare, T. F. , & Bahlman, S. H. (1971). The cardiovascular effects of a new inhalation anesthetic, Forane, in human volunteers at constant arterial carbon dioxide tension. Anesthesiology, 35, 8–16. 4932622 10.1097/00000542-197107000-00005

[brb3833-bib-0035] Zaretsky, A. , & Shofti, R. (2007). Comparison between the canine and caprine model for chronic vagus nerve electrical stimulation. Procedures, 7th Annual Scientific Meeting of ESLAV. Cernobio: European Society of Laboratory Animal Veterinarians.

